# Cholera

**Published:** 1866-02

**Authors:** 


Should the cholera come next summer, it will not be felt that we have said one word too much. We desire further to say, that as it may come in a night, and with great malignity, may fall on multitudes of families between the setting and the rising of the sun, and all cannot command a physician, it is the part of wisdom as well as the duty of all, especially of heads of families, to remember that there is a probability that physicians must be extemporized for the occasion, and fortunate will they be who had treasured up some practical knowledge on the subject; for it will enable them to save many valuable lives, if they only make themselves masters of the external treatment, and to administer internal remedies, only in case no physician can be had. As to the external treatment, the easiest and simplest of all, until a medical man can be brought, two additional facts may be stated. Of all the physicians in Great Britain during the last cholera, Dr. Ayre was perhaps the most successful and the most celebrated. A French gentleman left an amount of money to be given to the writer of the best essay on the nature and treatment of the disease. Of all the articles handed in, the one which met with the most decided approbation was that which was founded on Dr. Ayre's practice, which was to rely on calomel. If, then, calomel has had such strong advocates in America, in England, in France, and above all in India, it must have positive merit up to this time; hence, the probabilities are in its favor, of its future efficacy. Physicians of all shades are earnestly invited to direct their attention to this important practical point. The reason for the efficacy of calomel is simply this. Cholera is essentially the result of the liver failing to separate the bile from the blood; in medical phrase, it "secretes no bile," it is torpid; asleep; it does not work; scientific men, the world over, know that no medicine so certainly, so infalliably "acts on the liver," that is, "sets it going," makes it work, makes it separate the bile from the blood, and delivers it into the bowels as calomel; other medicines do this, but none of them with anything like the infallible certainty of calomel; it is the bile which gives color to the discharges from the bowels; in cholera they have no color; as soon as they do begin to have color, either yellow, green or black, depending on the quantity of bile, we know that the cholera patient begins to get well; and as the passages begin to loose their colorless appearance, they lessen in frequency and become thicker, more consistent; then, another wheel of the human machinery begins to turn, that is, the kidneys begin to work; the patient begins to urinate and he is safe! No man ever did get well of actual cholera without these changes; the first effect of an efficient dose of calomel in cholera is the passages are less frequent within two hours; then more colored, then more consistent, and the crisis is past. Whatever causes fevers ordinarily, will cause cholera in cholera times. Whatever depresses the mind in cholera times is a cause of an actual attack in individual cases; hence, worriement, fear, despondency, will bring on the disease. Whatever weakens the body will cause cholera, whether it be overdoing or any debilitating ailment. If you wait until the cholera appears around you, flight is fear, and fear is death; you carry the seeds of the disease with you, and place yourself more completely beyond the possibility of skilful medical aid; and even if you can get it, the difference between a stranger's interest and your own family physician is infinite odds against you. To every family in New-York, or other large cities, who doubts its courage, we say on the 1st of May next go to some country house in Northern New-England, especially Vermont, and remain there *altogether* if you have the means; for the flatter and the warmer a country is the more it is liable to the scourge; and by going before the warm weather disseminates the seeds of the disease, you will in proportion be safe from an attack. Dr. Patterson, of the Egyptian Medical Service, in a letter to the "London Medical Times and Gazette" says that the cholera of 1865 was of such a virulent form at Cairo and along the Mediterranean that the premonitory symptoms of diarrhœa were not present, but that men and women in the prime of life and in apparent robust health were struck down with it and died in from eight to twelve hours, either all the functions of life were suddenly suspended or instantaneous vomiting and purging took place, or cramps came on in the beginning. In all cases the engorgement of the liver was present, and in cases of recovery large quantities of a black oily substance was discharged, which was acrid bile, showing conclusively, as above stated, that the essence of the disease is the inaction of the liver, and that when it begins to work, the patient begins to improve and urination and recovery follow. It is scarcely possible, if it ever appears among us, that it will manifest itself in such a virulent form, because the filth and squalid poverty and depraved constitutions, from laziness and vicious habits of life, are not to be found in this country, which are almost universal among the semi-barbarous peoples of those warm countries. But as physicians will be the first to discover any peculiar phases of the disease, it will be the part of wisdom in all cases to secure the services of a medical man at the very earliest possible moment.

